# Prediction of Clinical Severity of COVID‐19 Using a Combination of Heparin‐Binding Protein, Interleukin‐6, and C‐Reactive Protein: A Retrospective Study

**DOI:** 10.1111/crj.70003

**Published:** 2024-08-26

**Authors:** Yidan Gao, Ke Zhao, Jing Liu, Xiangbo Zhang, Ling Gong, Xiang Zhou, Gongying Chen

**Affiliations:** ^1^ Department of Hepatology The Affiliated Hospital of Hangzhou Normal University Hangzhou Zhejiang China; ^2^ Department of Immunology and Pathogen Biology, School of Basic Medical Sciences Hangzhou Normal University Hangzhou Zhejiang China

**Keywords:** COVID‐19, C‐reactive protein, heparin‐binding protein, interleukin‐6, prognostic marker, SARS‐CoV‐2

## Abstract

**Background:**

Systemic inflammation stands as a pivotal factor tightly interwoven with the progression of COVID‐19. This study endeavors to elucidate the significance of three key inflammatory molecules, that is, heparin‐binding protein (HBP), interleukin‐6 (IL‐6), and C‐reactive protein (CRP), in assessing the severity and prognostic implications of COVID‐19.

**Methods:**

The demographic, clinical, and laboratory data were retrospectively collected from a cohort of 214 adult patients diagnosed with COVID‐19. Patients were divided into two groups: nonsevere (*n* = 93; 43.5%) and severe (*n* = 121; 56.5%). Additionally, based on their organ function, patients were categorized into nonorgan failure (*n* = 137) and organ failure (*n* = 77) groups. The levels of inflammation‐related cytokines were then compared among these defined groups.

**Results:**

The severe group was characterized by a higher proportion of males, older age, and longer hospital stays compared to nonsevere cases. Additionally, severe cases exhibited a higher prevalence of underlying diseases and organ failure. Statistical analysis revealed significantly elevated levels of HBP, IL‐6, and CRP in the severe group. HBP, IL‐6, and CRP were identified as independent risk factors for severe COVID‐19. Furthermore, a combined assessment of these biomarkers demonstrated superior diagnostic sensitivity (85.10%) and specificity (95.70%) for predicting COVID‐19 severity. A positive relationship between elevated HBP, IL‐6, and CRP levels and impaired organ function was also observed. The predictive efficiency significantly increased (hazard ratio = 3.631, log‐rank *p* = 0.003) when two or more of them were combinedly used. Notably, elevated levels of HBP, IL‐6, and CRP were associated with an increased risk of mortality.

**Conclusions:**

In conclusion, the combined assessment of HBP, IL‐6, and CRP offers enhanced accuracy and specificity in predicting the severity, organ failure, and mortality risk associated with COVID‐19.

## Graphical abstract

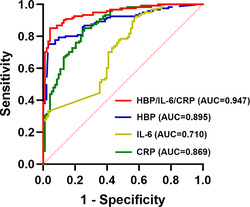

## 1 Introductions

Late in 2019, the emergence of COVID‐19, caused by the novel coronavirus SARS‐CoV‐2, led to a swift global spread, prompting the World Health Organization (WHO) to declare it a pandemic in March 2020 [1, 2]. During the pandemic, the virus continues to evolve, leading to various changes in its pathogenicity, immune evasion, and infectivity [3]. COVID‐19 patients typically present a broad spectrum of clinical manifestations, ranging from asymptomatic or mild illness to severe outcomes [2, 4]. Importantly, severe COVID‐19 often leads to respiratory failure, multiorgan dysfunction, thromboembolic complications, and even death [4]. In pregnant women with severe COVID‐19, rates of cesarean delivery and iatrogenic prematurity were also increased [5]. In addition, patients recovering from severe COVID‐19 may experience long‐term health complications, such as persistent respiratory symptoms, cognitive impairment, or postintensive care syndrome (PICS) [6]. Maintaining the healthcare system's capacity to meet the needs of critically ill patients is a significant challenge during the pandemic. As of February 2024, the global impact of COVID‐19, with over 774 million reported cases and more than 7 million deaths worldwide, underscores the severity and magnitude of the pandemic [1].

Early identification of COVID‐19 patients at risk of severe illness allows clinicians to intervene promptly, potentially improving outcomes and reducing mortality rates. Several significant risk factors have been identified for poor prognosis in SARS‐CoV‐2 infection, including advanced age, male gender, underlying health conditions, and immunocompromised states [7, 8]. In addition, compared to the original strain of SARS‐CoV‐2, certain variants like Delta and Omicron demonstrate altered virulence and heightened transmissibility, which may compromise the efficacy of immune responses and increase disease severity [9]. On the other hand, many studies have indicated that vaccination against the SARS‐CoV‐2 virus is one of the primary protective factors affecting mortality and clinical severity of COVID‐19 [10, 11]. Nevertheless, predicting the severity of COVID‐19 is a highly intricate process, demanding the integration of those risk and protective factors, none of which serve as universally reliable predictors [12, 13, 14, 15]. Furthermore, COVID‐19 can deteriorate rapidly, prompting the need for frequent reassessment and continuous refinement of predictive models in clinical practice. Despite ongoing efforts to enhance the prediction of severe COVID‐19 cases, it remains a challenging task due to the multifactorial nature of the disease.

Notably, recent studies have elucidated the pivotal role of systemic inflammation in driving the progression of COVID‐19 [15, 16, 17, 18]. Systemic inflammation is often characterized by a cytokine storm, where the immune system releases excessive levels of proinflammatory cytokines and molecules in response to the viral infection [18, 19, 20]. The resultant dysregulated immune response can lead to tissue damage, organ dysfunction, and exacerbation of the disease, making it a primary contributor to severe COVID‐19 [19, 20]. More importantly, several inflammatory molecules have been identified as independent predictors of severe COVID‐19 [15, 16, 21, 22, 23, 24, 25, 26, 27]. For example, heparin‐binding protein (HBP), a protein predominantly found in neutrophil granules and released during inflammatory responses, has shown potential as a predictor of mortality in COVID‐19 patients [21, 22]. Likewise, interleukin‐6 (IL‐6), a pleiotropic cytokine produced by a variety of cell types, has become increasingly recognized as a key player in COVID‐19 pathogenesis [22, 23, 24, 25]. Moreover, IL‐6 has been linked to severe lung injury and organ dysfunction in COVID‐19 patients, thus functioning as a predictor of disease severity [14, 25]. In addition, the level of C‐reactive protein (CRP), a crucial indicator of inflammation, has been found to be significantly elevated in severe cases of COVID‐19 [26, 27]. While cytokine levels can indicate an inflammatory response, their variability and overlap with other conditions reduce their reliability as standalone predictive markers for COVID‐19 severity. Consequently, relying solely on individual molecules for predicting the course of COVID‐19 remains insufficient, and whether the diagnostic efficiency of predicting COVID‐19 severity can be improved by the combined assessment of HBP, IL‐6, and CRP is currently unknown.

Therefore, the aim of this study is to investigate the predictive value of the combination of HBP, IL‐6, and CRP for assessing the severity of COVID‐19. By integrating these inflammation markers into a unified predictive model, we aim to enhance the accuracy and reliability of severity prediction, ultimately guiding more effective clinical decision‐making and improving COVID‐19 patient outcomes.

## 2 Methods

### 2.1 Study Design and Participants

This single‐center, retrospective cohort study included 214 adult patients diagnosed with COVID‐19, confirmed by RT‐PCR testing for SARS‐CoV‐2 infection, from December 1, 2022, to February 28, 2023. The demographic, clinical, and laboratory data of all patients were tracked and obtained through the in‐hospital electronic health information system. Patients who had hematological tumors, immune deficiency diseases, or recent use of glucocorticoids or immunosuppressants were excluded. The severity of COVID‐19 was evaluated based on “the 10th Version of the Novel Coronavirus Pneumonia Diagnosis and Treatment Guidance issued by the National Health Commission of China” [28].

According to the guideline, severe COVID‐19 was defined as meeting any of the following criteria and excluding interpretations due to reasons unrelated to COVID‐19 infection: (1) onset of dyspnea, with a respiratory rate (RR) ≥ 30 breaths/min; (2) resting oxygen saturation (SpO_2_) ≤ 93% while breathing ambient air; (3) arterial oxygen partial pressure/oxygen concentration ≤ 300 mmHg (1 mmHg = 0.133 kPa); and (4) progressive worsening of clinical symptoms, with radiographic evidence of lung lesion progression exceeding 50% within 24–48 h. The nonsevere group patients met the following conditions: (1) mild clinical symptoms; (2) persistent high fever lasting >3 days and/or cough, dyspnea, etc., with RR < 30 breaths/min and resting SpO_2_ ≤ 93% while breathing ambient air; and (3) characteristic radiographic manifestations of COVID‐19 pneumonia are observed.

Organ failure included respiratory failure, heart failure, liver failure, and renal failure. Diagnoses of organ failure were based on the following criteria: “ELSO Guidelines for Adult Respiratory Failure” [29], “Chinese guidelines for the diagnosis and treatment of heart failure 2024” [30], “Guideline for diagnosis and treatment of liver failure” [31], and “Chinese clinical practice guideline for acute kidney injury” [32]. In brief, respiratory failure was diagnosed according to arterial oxygen partial pressure ≤ 60 mmHg and/or partial pressure of carbon dioxide (PaCO2) ≥ 50 mmHg while breathing ambient air, and cases with intracardiac anatomical shunting and primary reductions in cardiac output were excluded. Heart failure was diagnosed primarily based on N‐terminal proBNP (NT‐proBNP) levels: > 450 ng/L for patients under 50 years old; > 900 ng/L for those over 50 years old; and > 1800 ng/L for those over 75 years old. Liver failure was generally defined by severe gastrointestinal symptoms, coagulopathy (prothrombin activity < 40% or international normalized ratio [INR] ≥ 1.5), elevated liver biochemistry (increase in total serum bilirubin to 10 times or more than the upper limit of normal or increase in total serum bilirubin by ≥ 17.1 μmol/L/day), and hepatic encephalopathy without underlying chronic liver disease. Renal failure was diagnosed by the following criteria: an increase in serum creatinine (Cr) (Scr) to three times or more than the baseline, Cr ≥ 4.0 (353.6 μmol/L), a 75% reduction in glomerular filtration rate, or urine output (u/o) < 0.5 mL/kg/h for 12 h. Comorbidities refer to hypertension, diabetes, chronic liver disease, chronic kidney disease, chronic pulmonary disease, cardiovascular disease, nervous system disease, and solid tumors. Diagnoses of *comorbidities* were determined using “the 10th revision of the International Classification of Diseases (ICD‐10).” A thorough evaluation of medical records, diagnostic tests, and clinical assessments was accomplished by the multidisciplinary review team. This study was approved by the ethics committee of the Affiliated Hospital of Hangzhou Normal University.

### 2.2 Statistical Analysis

Statistical methods were selected according to the characteristics of the data. Categorical variates were analyzed using Fisher's exact test or the chi‐squared test, while continuous variates were analyzed using the Student *t*‐test or the Mann–Whitney *U* test. The diagnostic value was evaluated by the receiver operating characteristic (ROC) curve. The greatest Youden index was chosen as the optimal cut‐off value. Univariate and multivariate logistic regression models were further constructed to analyze the risk factors. The survival time was defined for survival analysis as the period from the admission date to the death date. The correlation between risk factors and clinical outcomes was assessed using the Kaplan–Meier (KM) test and the log‐rank test.

All analyses were implemented with SPSS 26.0, and GraphPad Prism 9 software was used for graphical representation. A *p* value < 0.05 suggested that the difference was statistically significant. For items beyond the scope of the inspection report, extreme values of the inspection report range were included in the statistical analysis.

## 3 Results

### 3.1 Comparison of Baseline Characteristics of the Study Population

A total of 214 adult patients diagnosed with COVID‐19 were enrolled in this study, including 129 men and 85 women. The baseline demographic, clinical, and laboratory data of the study population are summarized in Table S1. Among the cohort, 121 patients (56.5%) exhibited severe COVID‐19, with a median age of 83 years, while 93 patients (43.5%) were classified as nonsevere, with a median age of 67 years. Compared to the nonsevere group, the severe group involved more men than women (*p* = 0.002), older patients (*p* < 0.001), and longer hospital stays (*p* < 0.001). In addition, more underlying diseases were associated with severe COVID‐19. Concurrently, we also observed that 77 patients (36.0%) exhibited organ failure, with a median age of 83 years, whereas 137 patients (64.0%) did not experience organ failure, with a median age of 71 years (Table S1).

### 3.2 Risk Factors Associated With Severe COVID‐19

Notably, the severe group showed higher median levels of HBP (51.43 ng/mL), IL‐6 (70.10 pg/mL), and CRP (87.64 mg/mL) than the nonsevere group, and all differences were statistically significant (*p* < 0.001, Table S1). Univariate analysis indicated that HBP, IL‐6, CRP, gender (male), age, and comorbidities were significantly positively correlated with COVID‐19 severity (Table 1). By using step‐wise forward logistic regression analysis, the independent risk factors for the severe group were shown to be age (OR = 1.09, 95% CI: 1.05–1.13, *p* < 0.001), HBP (OR = 1.07, 95% CI: 1.04–1.11, *p* < 0.001), IL‐6 (OR = 1.01, 95% CI: 1.00–1.03, *p* = 0.030), and CRP (OR = 1.03, 95% CI: 1.01–1.04, *p* = 0.001).

**TABLE 1:** Univariate and multivariate logistic regression analyses of parameters related to the incidence of severe COVID‐19.

Variable	Nonsevere	Severe	Univariate analysis		Multivariate analysis model	
	*n* = 93	*n* = 121	OR (95% CIs)	*p*	OR (95% CIs)	*p*
Gender (male), *n* (%)	45 (48.39)	84 (69.42)	2.42 (1.38–4.25)	0.002		
Age (years), median (IQR)	67 (59–78)	83 (75–88)	1.08 (1.06–1.11)	< 0.001	1.08 (1.03–1.13)	0.002
Hypertension, *n* (%)	39 (41.94)	76 (62.80)	2.34 (1.35–4.07)	0.003		
Diabetes, *n* (%)	16 (17.20)	38 (31.40)	2.20 (1.13–4.27)	0.019		
Chronic kidney disease, *n* (%)	15 (16.12)	47 (38.84)	3.30 (1.70–6.41)	< 0.001		
Cardiovascular diseases, *n* (%)	26 (27.96)	62 (51.24)	2.71 (1.52–4.82)	0.001		
Nervous system disease, *n* (%)	8 (8.60)	37 (30.58)	4.68 (2.06–10.64)	< 0.001		
HBP (ng/mL), median (IQR)	16.35 (7.44–25.32)	51.43 (45.98–103.91)	1.09 (1.06–1.10)	< 0.001	1.08 (1.04–1.11)	< 0.001
IL‐6 (pg/mL), median (IQR)	24.09 (4.26–81.23)	70.10 (32.97–112.47)	1.02 (1.01–1.02)	< 0.001	1.01 (1.00–1.03)	0.030
CRP (mg/L), median (IQR)	16.63 (6.03–44.07)	87.64 (52.03–141.01)	1.03 (1.02–1.04)	< 0.001	1.03 (1.01–1.04)	0.001

Abbreviations: CIs, confidence intervals; CRP, C‐reactive protein; HBP, heparin‐binding protein; IL‐6, interleukin‐6; IQR, interquartile range; OR, odds ratio.

### 3.3 Combination of HBP, IL‐6, and CRP Had Higher Diagnostic Efficiency in Predicting Severe COVID‐19

**FIGURE 1:**
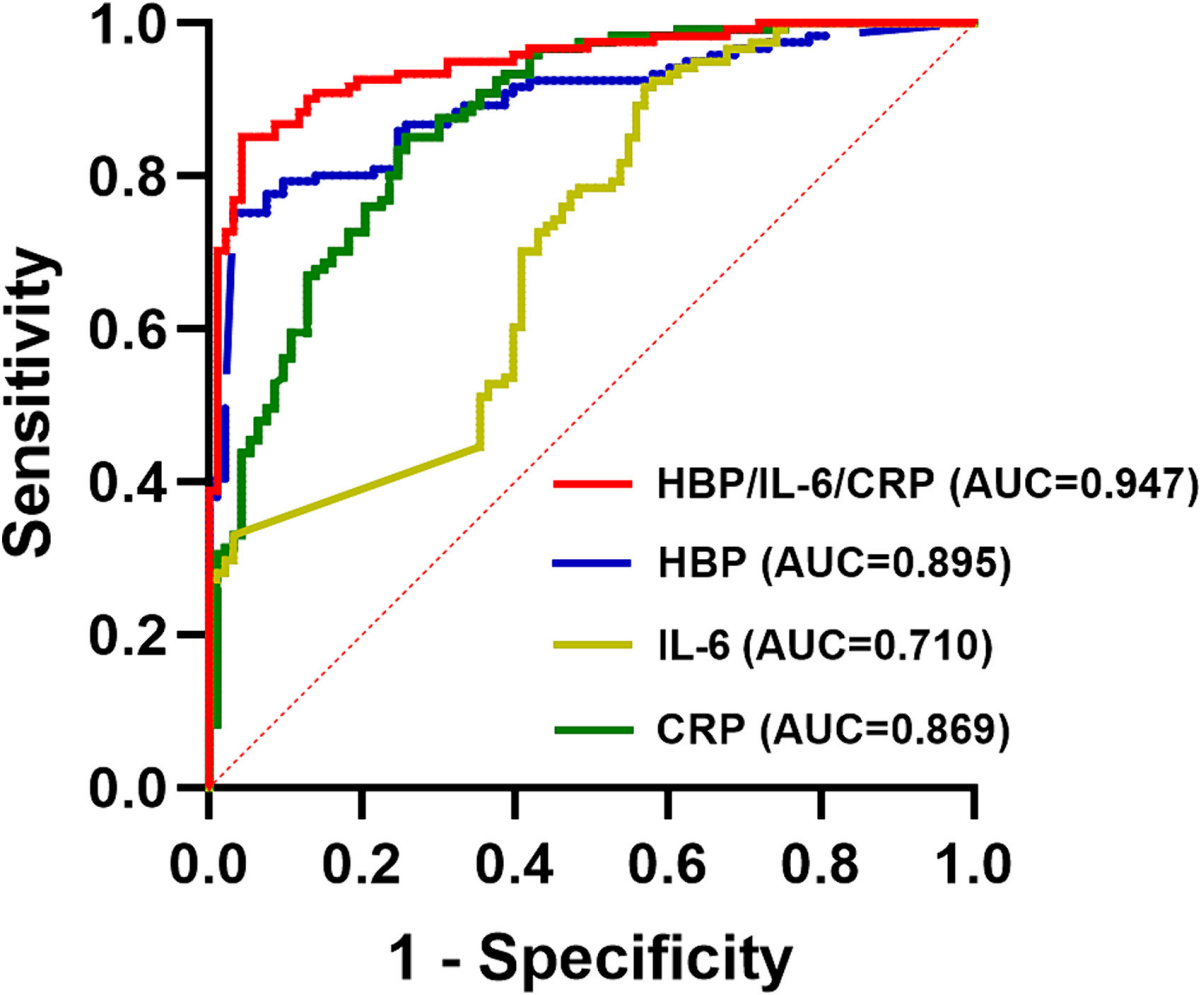
Receiver operating characteristic curves of HBP, IL‐6, CRP, and HBP/IL‐6/CRP combination to predict COVID‐19 severity. AUC, area under curve; HBP, heparin‐binding protein; IL‐6, interleukin‐6; CRP, C‐reactive protein.

The ROC curve was plotted using HBP, IL‐6, CRP, and HBP/IL‐6/CRP combination as test variables, with severe group classification serving as the state variable (Figure 1). The results suggested that the area under the curve (AUC) for predicting severe COVID‐19 using HBP, IL‐6, CRP, and HBP/IL‐6/CRP combination was 0.895 (95% CI: 0.853–0.938), 0.710 (95% CI: 0.641–0.780), 0.869 (95% CI: 0.821–0.917), and 0.947 (95% CI: 0.919–0.976), respectively. In addition, the HBP/IL‐6/CRP combination in the prediction of severe COVID‐19 had a sensitivity of 85.10%, a specificity of 95.70%, a positive predictive value of 96.30%, and a negative predictive value of 83.20% (Table 2). When the Youden index was at its maximum, the cut‐off values of HBP, IL‐6, and CRP were 49.71 ng/mL, 11.24 pg/mL, and 39.67 mg/L, respectively. Subsequently, a further grouping of our cohort was conducted based on the optimal cut‐off values as follows: Group 1 included patients with HBP ≤ 49.71 ng/mL, IL‐6 ≤ 11.24 pg/mL, and CRP ≤ 39.67 mg/L; Group 2 consisted of patients with only one of the three indicators exceeding the optimal cut‐off value; and Group 3 encompassed patients with two or more indicators surpassing the optimal cut‐off value.

**TABLE 2:** Analysis of receiver operating characteristic parameters to predict the severity of COVID‐19.

Parameters	AUC	95% CIs	Cut‐off	Sensitivity (%)	Specificity (%)	PPV (%)	NPV (%)	*p*
HBP	0.895	0.853–0.938	49.71	75.2	97.0	96.8	75.0	< 0.001
CRP	0.869	0.821–0.917	39.67	85.1	74.2	81.1	79.3	< 0.001
IL‐6	0.710	0.641–0.78	11.24	91.7	43.0	67.7	80.0	< 0.001
HBP/IL‐6/CRP	0.947	0.919–0.976	0.62	85.1	95.7	96.3	83.2	< 0.001

Abbreviations: CIs, confidence intervals; CRP, C‐reactive protein; HBP, heparin‐binding protein; IL‐6, interleukin‐6; NPV, negative predictive value; PPV, positive predictive value.

### 3.4 Correlation Between HBP, IL‐6, and CRP Levels and Indexes of Organ Function

**FIGURE 2:**
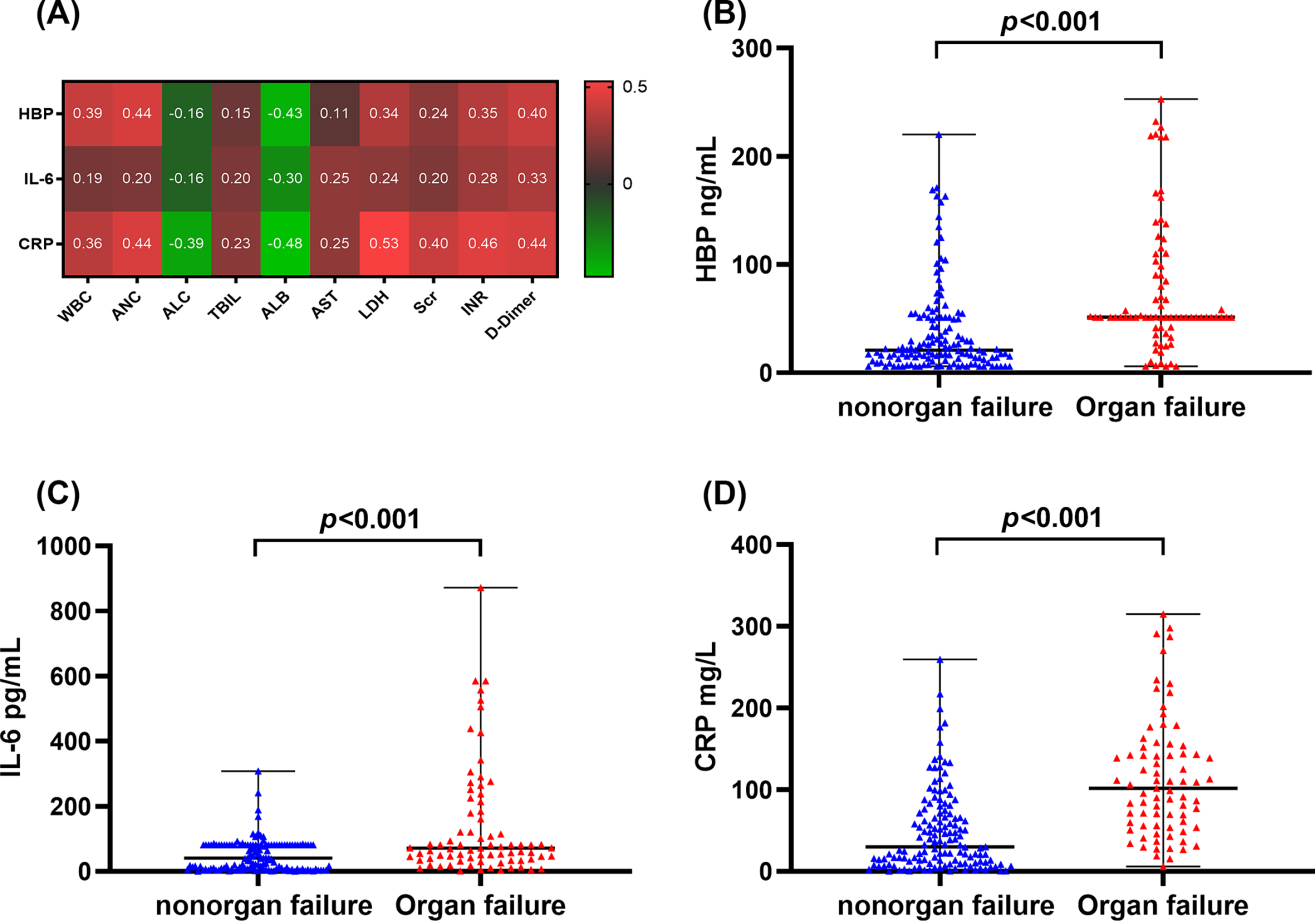
The relationship between inflammatory indicators and the organ failure of COVID‐19. (A) Spearman's rank correlation analysis of HBP, IL‐6, and CRP and indexes of organ function. Comparison of (B) HBP, (C) IL‐6, and (D) CRP between nonorgan failure and organ failure groups. *p* values for the Mann–Whitney *U* test of comparison were displayed. Quantitative data are represented by mean ± standard deviation (SD). HBP, heparin‐binding protein; IL‐6, interleukin‐6; CRP, C‐reactive protein; WBC, white blood cell count; ANC, absolute neutrophil count; ALC, absolute lymphocyte count; TBIL, total bilirubin; ALB, albumin; AST, aspartate aminotransferase; LDH, lactate dehydrogenase; Scr, serum creatinine; INR, international normalized ratio.

The Spearman rank correlation analysis suggested that the levels of HBP, IL‐6, and CRP were significantly positively correlated with total bilirubin (TBIL), lactate dehydrogenase (LDH), Scr, INR, and D‐dimer, and were significantly negatively correlated with albumin (ALB) (Figure 2A). Besides, the levels of IL‐6 and CRP exhibited significant positive correlations with aspartate aminotransferase (AST), while no significant correlation was observed between HBP and AST levels (Figure 2A).

### 3.5 Risk Factors Associated With COVID‐19 Patients Experiencing Organ Failure

The organ failure group showed higher median levels of HBP (51.43 ng/mL), IL‐6 (71.57 pg/mL), and CRP (101.63 mg/mL) than the nonorgan failure group, and all differences were statistically significant (*p* < 0.001, Figure 2B–D, Table S1). Univariate analysis revealed that HBP, IL‐6, CRP, HBP/IL‐6/CRP combination, age, and comorbidities are significantly positively correlated with the occurrence of organ failure (Table 3). After adjustment for age and comorbidities at baseline, HBP, IL‐6, and CRP were used as independent variables (Model 1; Table 3). By using step‐wise forward logistic regression analysis, the independent risk factors for organ failure in COVID‐19 patients were identified as follows: age (OR = 1.07, 95% CI: 1.03–1.11, *p* = 0.001), CVD (OR = 2.74, 95% CI: 1.13–6.14, *p* = 0.140), HBP (OR = 1.01, 95% CI: 1.00–1.02, *p* = 0.018), IL‐6 (OR = 1.01, 95% CI: 1.00–1.01, *p* = 0.005), and CRP (OR = 1.02, 95% CI: 1.01–1.02, *p* < 0.001). When maintaining the baseline unchanged and considering the HBP/IL‐6/CRP combination as the independent variable (Model 2; Table 3), the independent risk factors for organ failure were shown to be age (OR = 1.05, 95% CI: 1.02–1.08, *p* = 0.003), CVD (OR = 2.34, 95% CI: 1.14–4.80, *p* = 0.021), and HBP/IL‐6/CRP combination (*p* < 0.001). Notably, patients in Group 3 had the highest risk of organ failure compared to Group 1 (OR = 9.47, 95% CI: 1.99–45.13, *p* = 0.005) and Group 2 (OR = 10.00, 95% CI: 3.50–28.54, *p* < 0.001).

**TABLE 3:** Univariate and multivariate logistic regression analyses of parameters related to the incidence of organ failure.

Variable	Nonorgan failure	Organ failure	Univariate analysis		Multivariate analysis Model 1		Multivariate analysis Model 2	
	*n* = 137	*n* = 77	OR (95% CIs)	*p*	OR (95% CIs)	*p*	OR (95% CIs)	*p*
Age (years), median (IQR)	71 (61–84)	83 (77–89)	1.08 (1.04–1.10)	< 0.001	1.07 (1.03–1.11)	0.001	1.05 (1.02–1.08)	0.003
Diabetes, *n* (%)	26 (17.20)	28 (31.40)	2.44 (1.30–4.58)	0.006				
Chronic kidney disease, *n* (%)	27 (18.98)	35 (45.45)	3.40 (1.84–6.28)	< 0.001				
Cardiovascular disease, *n* (%)	41 (29.93)	47 (61.04)	2.67 (2.04–6.59)	0.001			2.34 (1.14–4.80)	0.021
Nervous system disease, *n* (%)	21 (15.33)	24 (31.17)	2.50 (1.28–4.89)	0.007				
HBP (ng/mL), median (IQR)	21.00 (11.97–51.43)	51.43 (46.81–101.04)	1.02 (1.01–1.02)	< 0.001	1.01 (1.00–1.02)	0.018		
IL‐6 (pg/mL), median (IQR)	40.88 (8.13–81.23)	71.57 (35.68–181.72)	1.01 (1.01–1.02)	< 0.001	1.01 (1.00–1.01)	0.005		
CRP (mg/L), median (IQR)	30.09 (10.15–71.00)	101.63 (55.79–147.93)	1.02 (1.01–1.02)	< 0.001	1.02 (1.01–1.02)	< 0.001		
HBP/IL‐6/CRP				< 0.001				< 0.001
Group 2 vs. 1								
Group 3 vs. 1			16.29 (3.72–71.42)	< 0.001			9.47 (1.99–45.13)	0.005
Group 3 vs. 2			12.55 (4.70–33.49)	< 0.001			10.00 (3.50–28.54)	< 0.001

*Note:* Model 1 was adjusted for age, diabetes, chronic kidney disease, cardiovascular disease, and nervous system disease at baseline, with HBP, IL‐6, and CRP as independent variables. Model 2: the baseline remained unchanged, with the HBP/IL‐6/CRP combination as the independent variable.

Abbreviations: CIs, confidence intervals; CRP, C‐reactive protein; HBP, heparin‐binding protein; IL‐6, interleukin‐6; IQR, interquartile range; OR, odds ratio.

### 3.6 Combination of HBP, IL‐6, and CRP Had Higher Accuracy in Predicting the Risk of Death Caused by COVID‐19

**FIGURE 3:**
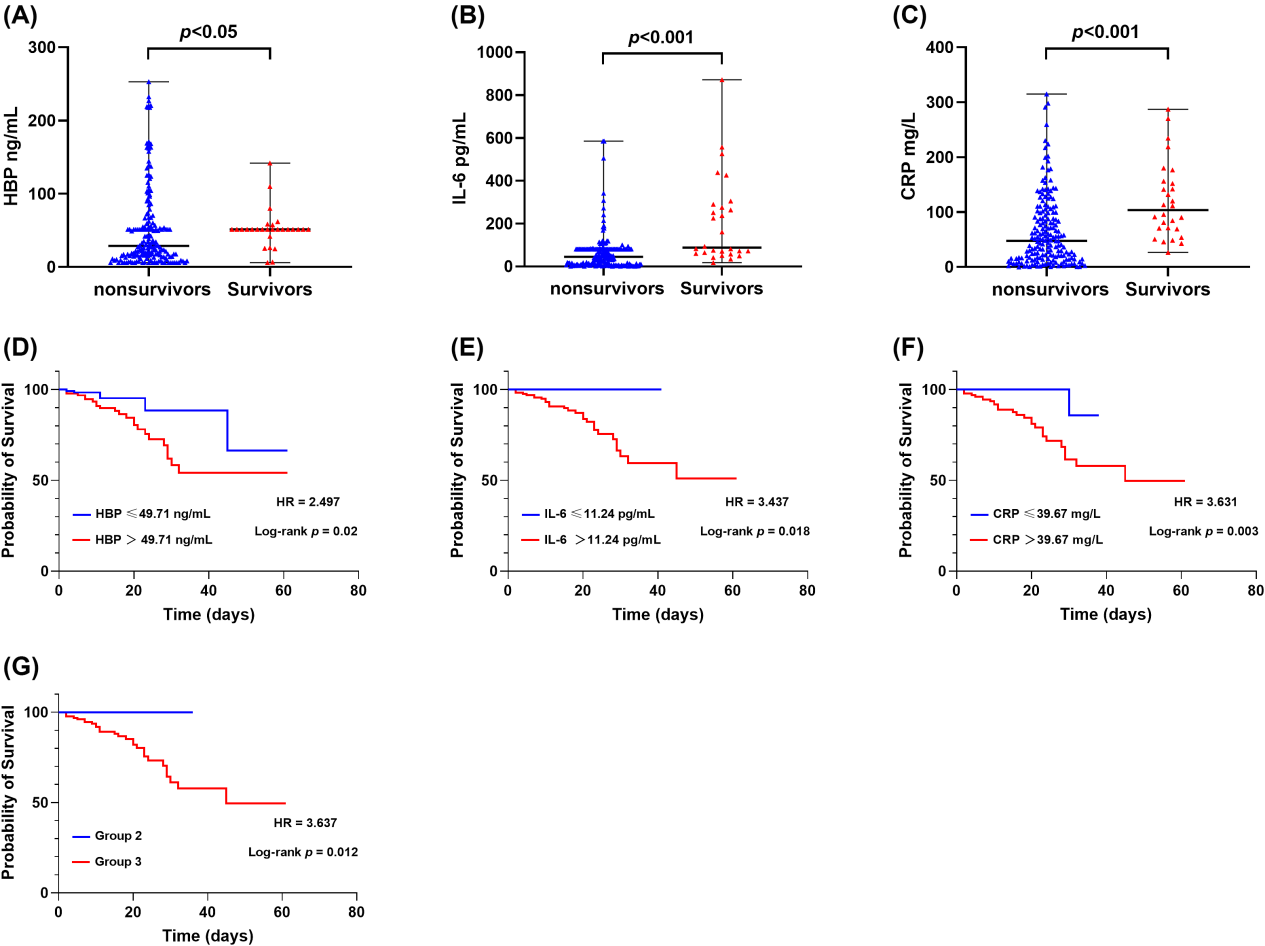
HBP, IL‐6, CRP, and HBP/IL‐6/CRP combination predicted the mortality of COVID‐19. Comparison of HBP (A), IL‐6 (B), and CRP (C) between nonsurvivor and survivor groups. *p* values for the Mann–Whitney *U* test of comparison were displayed. Quantitative data are represented by mean ± standard deviation (SD). Kaplan–Meier survival curves were displayed according to the cut‐off values of HBP (D), IL‐6 (E), CRP (F), and the different groups dived by HBP/IL‐6/CRP combination (G). *p* values and the hazard ratios (HRs) were presented.

The nonsurvivor group exhibited significantly higher median levels of HBP, IL‐6, and CRP compared to the survivor group, with all differences being statistically significant (*p* < 0.001, Figure 3A–C). KM survival curves revealed that HBP (HR = 2.497, log‐rank *p* < 0.001), IL‐6 (HR = 3.437, log‐rank *p* = 0.018), and CRP (HR = 3.631, log‐rank *p* = 0.003) levels exceeding the optimal cut‐off values were associated with a markedly increased risk of mortality (Figure 3D–G). Moreover, Group 3 exhibited a higher mortality risk compared to Group 2 (3 vs. 2, HR = 3.631, log‐rank *p* = 0.003).

## 4 Discussion

SARS‐CoV‐2, the virus responsible for COVID‐19, primarily infiltrates host cells by binding to the angiotensin‐converting enzyme 2 (ACE2) receptor, which is highly expressed in various tissues such as the respiratory epithelium, cardiovascular system, kidneys, and gastrointestinal tract [33, 34]. Upon entry, the virus triggers an immune response involving the activation of innate immune cells like macrophages and dendritic cells [34]. These cells recognize viral components through pattern recognition receptors (PRRs) such as Toll‐like receptors (TLRs) and retinoic acid–inducible gene I (RIG‐I)‐like receptors (RLRs) [33, 34]. This recognition prompts the production of proinflammatory cytokines and Type I interferons (IFNs), establishing an antiviral state and attracting immune cells to the infection site [34]. On the one hand, the cytopathic effect of SARS‐CoV‐2 can directly cause cell death and tissue damage, increasing the infection's severity [34]. Additionally, the substantial infiltration of immune cells can worsen tissue damage by releasing reactive oxygen species (ROS), proteases, and more proinflammatory cytokines [34]. Therefore, a well‐coordinated immune response is essential for combating the virus effectively while minimizing collateral damage to host tissues.

The systemic inflammation response plays a vital role in driving the progression of COVID‐19 [15, 16, 17, 18]. Inflammatory factors have long been regarded as important biomarkers reflecting the severity of COVID‐19. Elevated concentrations of those biomarkers signify a more intense cytokine‐mediated inflammatory response, which is closely linked to unfavorable outcomes in patients with COVID‐19 [15, 16, 21, 22, 23, 24, 25, 26, 27]. Even in populations with significant immunological changes, such as pregnant women, cytokine levels have shown significant positive correlations with severe COVID‐19 cases [35]. In our study, we observed a close correlation between the levels of HBP, IL‐6, and CRP and the severity, organ failure, and mortality associated with COVID‐19. Furthermore, the combination of HBP, IL‐6, and CRP proved to be effective in accurately predicting the severity and prognosis of COVID‐19.

On admission, the severe group exhibited higher levels of HBP, IL‐6, and CRP compared to the nonsevere group, which was consistent with previous findings indicating heightened inflammatory reactions in severe COVID‐19 cases [15, 16, 17]. Multivariate analysis identified these three indicators as independent risk factors for severe illness. The low odds ratios suggest that while the association between these indicators and COVID‐19 severity is statistically significant, it is not strong, potentially due to the small sample size of this study. Importantly, the HBP/IL‐6/CRP combination demonstrated higher sensitivity and specificity *in predicting disease severity*, surpassing individual detection methods. The optimal cut‐off values of HBP, IL‐6, and CRP for predicting COVID‐19 severity were broadly consistent with those reported in literatures. Previous studies proposed varying cut‐off values for these biomarkers, ranging from 13 to 18 ng/mL for HBP [22, 36], 24 to 32 pg/mL for IL‐6 [37, 38], and 20 to 97 mg/L for CRP [26, 27, 39]. While reports on HBP in COVID‐19 are relatively scarce, our study suggests that the combined assessment of these three molecules significantly improves the assessment of COVID‐19 severity.

In this study, we also observed a positive correlation between HBP, IL‐6, and CRP levels and markers of organ dysfunction such as TBIL, LDH, Scr, and D‐dimer. This indicates that HBP, IL‐6, and CRP may contribute to organ dysfunction in patients with COVID‐19, which is consistent with findings from previous reports [21, 25, 39]. Additionally, in critical conditions, inflammatory mediators will preferentially synthesize other acute phase reactants and reduce ALB synthesis [40]. Indeed, we noticed a negative association between HBP, IL‐6, CRP levels, and ALB, which was considered a reliable prognostic indicator for patients with severe COVID‐19 [40]. This insight enables early implementation of clinical interventions, thereby improving patient prognosis.

Based on the optimal cut‐off values determined earlier, our cohort can be divided into three groups. Group 1 comprised patients with three indicators all below the optimal cut‐off value; Group 2 included patients with only one of these three indicators exceeding the optimal cut‐off value; Group 3 consisted of patients with two or more indicators surpassing the optimal cut‐off value. We found that the admission levels of HBP, IL‐6, and CRP in the organ failure group were significantly higher than those in the nonorgan failure group. Logistic regression analysis further identified the HBP/IL‐6/CRP combination as an independent risk factor for organ failure. Notably, the predicted risk of organ failure was 9.47 times and 10 times higher in Group 3 compared to Groups 1 and 2, respectively, indicating a high probability of organ failure when any two or more biomarkers (HBP, IL‐6, and CRP) exceeded their respective optimal cut‐off values. Recent studies showed that COVID‐19 patients with elevated levels of HBP, IL‐6, and CRP faced a significantly increased risk of respiratory failure [21, 25, 39]. However, the risk of multiple organ failure in COVID‐19 associated with these biomarkers was not previously reported. Our study extends this understanding by showing that these biomarkers predict the overall risk of organ failure, encompassing respiratory, cardiac, hepatic, and other organ dysfunctions. Moreover, when considering the combined detection of HBP, IL‐6, and CRP, it demonstrated superior predictive value for the development of organ failure in COVID‐19 patients compared to individual factors.

Inflammatory storms have been closely associated with death in COVID‐19 patients [14, 15]. In this study, we revealed that nonsurvivors exhibited higher admission levels of HBP, IL‐6, and CRP compared to survivors. KM survival curves showed that patients with HBP, IL‐6, and CRP levels above the optimal cut‐off values had a considerably higher risk of mortality. Therefore, HBP, IL‐6, and CRP not only serve as indicators of multiple organ dysfunction syndrome and adverse outcomes in COVID‐19 but also hold the potential for predicting mortality. In fact, several studies have analyzed the predictive value of HBP, IL‐6, and CRP in assessing the death caused by COVID‐19. The reported cut‐off value for HBP was 35 ng/mL [22], while for IL‐6, values ranged from 24 to 83 pg/mL in different studies [24, 37, 41], and for CRP, they ranged from 20 to 151 mg/L [26, 27]. In contrast to previous reports, we used the combined detection of HBP, IL‐6, and CRP to predict the risk of death in COVID‐19. The risk of death predicted by any two or more indicators above the optimal cut‐off value was 3.6 times greater than that predicted by only one indicator above the optimal cut‐off value. Therefore, the combination of HBP, IL‐6, and CRP offers a more comprehensive evaluation of COVID‐19 prognosis, a novel finding not previously reported.

The systemic immune‐inflammation index (SII) and systemic inflammation response index (SIRI) offer straightforward and efficient methods for evaluating the inflammatory and immune status of patients through the integration of peripheral blood cell counts [42, 43]. In clinical practice, both indices serve as valuable prognostic indicators for various medical conditions, including cancer, cardiovascular diseases, infectious diseases, and autoimmune disorders. While several recent studies have revealed that elevated SII and SIRI values correlate with poor prognosis and adverse outcomes in COVID‐19 patients, this correlation remains a subject of ongoing debate [44, 45, 46, 47]. Compared to indices based on peripheral blood cell counts, inflammatory molecule profiles offer several advantages in predicting COVID‐19 severity. By measuring specific molecules associated with the pathophysiology of COVID‐19, such as IL‐6 and HBP, inflammatory molecule profiles provide more specific information about the immune dysregulation driving COVID‐19 severity, offering insights that generalized metrics like SII and SIRI cannot. Furthermore, inflammatory molecules implicated in COVID‐19 severity can serve as potential targets for therapeutic interventions. Monitoring those molecules can help identify patients who may benefit from targeted anti‐inflammatory therapies, such as IL‐6 inhibitors, to mitigate disease progression and improve outcomes.

## 5 Limitations

This is a single‐center, retrospective study, which may limit its generalizability to broader populations. Because of sample size limitations, we observed no significant difference in the risk of organ failure and mortality between Group 1 and Group 2 by the HBP/IL‐6/CRP combination. In addition, a small sample size may also contribute to the attenuation of the odds ratios when assessing the association of these biomarkers with COVID‐19 severity. Moreover, as our study focused on hospitalized COVID‐19 patients, there may be inherent population bias, potentially reducing the statistical significance of our findings. Including outpatient patients in future studies could enhance the robustness and relevance of our results. Meanwhile, the baseline demographic characteristics, particularly concerning sex and age, in severe and nonsevere COVID‐19 groups can potentially influence the study parameters and subsequent analyses.

## 6 Conclusions

In summary, the combined assessment of HBP, IL‐6, and CRP offers enhanced accuracy and specificity in predicting the severity, organ failure, and mortality risk associated with COVID‐19. Utilizing these biomarkers in combination can provide valuable prognostic information for severe COVID‐19 cases, enabling timely intervention to reduce mortality and improve patient outcomes.

## Author Contributions

Conceptualization: Yidan Gao, Jing Liu, and Gongying Chen. Formal analysis: Yidan Gao and Ke Zhao. Investigation: Xiangbo Zhang. Methodology: Ke Zhao. Writing – original draft: Yidan Gao and Ke Zhao. Writing – review and editing: Ling Gong, Xiang Zhou, and Gongying Chen. All authors have read and agreed to the published version of the manuscript.

## Ethics Statement

The Affiliated Hospital of Hangzhou Normal University's Ethics Committee granted clearance for this study according to the Declaration of Helsinki, with approval number 2023(E2)‐KS‐093. The Affiliated Hospital of Hangzhou Normal University's Ethics Committee exempted informed consent for the study as it was a retrospective study conducted through observation. All methods were carried out in accordance with relevant guidelines and regulations.

## Conflicts of Interest

The authors declare no conflicts of interest.

## Supporting information


**Table S1.** Baseline and clinical traits of patients correlated with the occurrence of severe COVID‐19 and organ failure.

## Data Availability

The datasets analyzed during the current study are available from the corresponding author on reasonable request.
