# Supplementary material for: Prediction of Clinical Severity of COVID‐19 Using a Combination of Heparin‐Binding Protein, Interleukin‐6, and C‐Reactive Protein: A Retrospective Study
Source: Clin Respir J. 2024 Aug 26;18(8):e70003. doi: 10.1111/crj.70003 (PMC11347126; doi:10.1111/crj.70003)
Supplement: Supplementary file 1 — Table S1. Baseline and clinical traits of patients correlated with the occurrence of severe COVID‐19 and organ failure. [file CRJ-18-e70003-s001.docx]

**Supplementary Table 1** Baseline and clinical traits of patients correlated with the occurrence of severe COVID-19 and organ failure.

|  | **Non-severe** | **Severe** | ***P***  **value** | **Non-organ failure** | **Organ failure** | ***P***  **value** |
| --- | --- | --- | --- | --- | --- | --- |
|  | **(n=93)** | **(n=121)** |  | **(n=137)** | **(n=77)** |  |
| **Demographic characteristics** | | | | | | |
| Gender (n, %) |  |  | 0.002 |  |  | 0.193 |
| Male | 45(48.39) | 84(69.42) |  | 78(56.94) | 51(66.23) |  |
| Female | 48(51.61) | 37(30.58) |  | 59(43.07) | 26(33.77) |  |
| Age, years,  median (IQR) | 67.00(58.50-77.50) | 83.00(74.5-88.0) | 0.000 | 71.00(61.00-84.50) | 83.00(76.50-89.00) | 0.000 |
| BMI, kg/m2,  median (IQR) | 23.73(20.93-25.95) | 23.70(21.36-24.57) | 0.323 | 23.70(21.31-25.95) | 23.70(21.08-23.87) | 0.091 |
| **Laboratory results, median (IQR)** | | | | | | |
| WBC, x10^9/L | 4.79(3.73-6.87) | 6.68(5.02-9.12) | 0.000 | 5.23(3.89-7.05) | 7.66(5.58-11.36) | 0.000 |
| ANC, x10^9/L | 3.61(2.35-4.97) | 5.18(3.96-8.10) | 0.000 | 3.90(2.44-5.29) | 6.19(4.48-9.83) | 0.000 |
| ALC, x10^9/L | 0.93(0.74-1.41) | 0.63(0.38-0.98) | 0.000 | 0.90(0.66-1.31) | 0.49(0.30-0.82) | 0.000 |
| HB, g/L | 124.00(111.00-137.00) | 118(101.50-131.50) | 0.009 | 125.00(110.50-137.00) | 117.00(98.00-129.00) | 0.003 |
| Platelet, x10^9/L | 179.72(141.50-238.50) | 158.00(107.50-209.99) | 0.020 | 176.00(133.50-226.50) | 156(89.50-212.00) | 0.019 |
| PCT, ng/mL | 0.17(0.11-1.84) | 0.24(0.11-1.84) | 0.348 | 0.18(0.11-1.84) | 0.41(0.13-1.84) | 0.019 |
| HBP, ng/mL | 16.35(7.44-25.32) | 51.43(45.98-103.91) | 0.000 | 21.00(11.97-51.43) | 51.43(46.81-101.04) | 0.000 |
| IL-6, pg/mL | 24.09(4.26-81.23) | 70.10(32.97-112.47) | 0.000 | 40.88(8.13-81.23) | 71.57(35.68-181.72) | 0.000 |
| CRP, mg/L | 16.63(6.03-44.07) | 87.64(52.03-141.01) | 0.000 | 30.09(10.15-71.00) | 101.63(55.79-147.93) | 0.000 |
| Fibrinogen, g/L | 3.95(3.28-4.79) | 4.49(3.51-5.59) | 0.017 | 4.16(3.51-4.86) | 4.61(3.29-5.59) | 0.227 |
| INR | 1.06(1.02-1.12) | 1.12(1.08-1.21) | 0.000 | 1.08(1.03-1.12) | 1.14(1.10-1.23) | 0.000 |
| D-dimer, mg/L | 0.40(0.26-0.77) | 1.27(0.66-3.45) | 0.000 | 0.50(0.31-0.90) | 2.27(0.90-4.59) | 0.000 |
| TBIL, μmol/L | 11.40(8.90-14.30) | 13.70(10.20-17.45) | 0.005 | 11.50(9.30-15.10) | 13.90(10.25-17.95) | 0.044 |
| ALT, U/L | 23.00(16.00-38.50) | 25.00(16.00-40.30) | 0.484 | 23.00(16.00-40.60) | 23.00(14.00-38.00) | 0.223 |
| AST, U/L | 29.00(21.00-39.50) | 37.00(25.00-54.50) | 0.000 | 30.00(22.00-44.00) | 40.00(25.50-56.40) | 0.014 |
| ALB, g/L | 37.40(34.28-40.95) | 31.60(28.60-34.25) | 0.000 | 35.80(32.90-39.70) | 30.90(26.95-33.94) | 0.000 |
| Scr, μmol/L | 65.40(56.90-81.65) | 88.00(54.90-123.65) | 0.000 | 70.40(58.60-90.00) | 95.40(63.15-129.65) | 0.000 |
| LDH, U/L | 218.00(184.00-258.50) | 308.89(238.00-400.00) | 0.000 | 232.00(190.50-286.00) | 332.00(274.00-446.00) | 0.000 |
| Troponin, ng/mL | 0.03(0.02-0.22) | 0.08(0.02-0.22) | 0.138 | 0.03(0.02-0.22) | 0.08(0.03-0.22) | 0.003 |
| **Comorbidities, n (%)** | | | | | | |
| Hypertension | 39(41.94) | 76(62.81) | 0.004 | 67(48.91) | 48(62.34) | 0.065 |
| Diabetes | 16(17.20) | 38(31.40) | 0.026 | 26(18.98) | 28(36.36) | 0.008 |
| Chronic liver disease | 32(34.41) | 42(34.71) | 1.000 | 48(35.04) | 26(33.77) | 0.882 |
| Chronic kidney disease | 15(16.13) | 47(38.84) | 0.000 | 27(19.71) | 35(45.45) | 0.000 |
| Chronic pulmonary disease | 11(11.83) | 4(3.31) | 0.028 | 10(7.30) | 5(6.49) | 1.000 |
| Cardiovascular disease | 26(27.96) | 62(51.24) | 0.001 | 41(29.93) | 47(61.04) | 0.000 |
| Nervous system disease | 8(8.60) | 37(30.58) | 0.000 | 21(15.33) | 24(31.17) | 0.009 |
| Solid tumors | 17(18.28) | 15(12.40) | 0.251 | 24(17.52) | 8(10.39) | 0.230 |

BMI, body mass index; IQR, interquartile range; WBC, white blood cell count; ANC, absolute neutrophil count; ALC, absolute lymphocyte count; HB, hemoglobin; PCT, procalcitonin; HBP, heparin-binding protein; IL6, interleukin-6; CRP, C-reactive protein; INR, international normalized ratio; TBIL, total bilirubin; ALT, alanine amiotransferase; AST, aspartate aminotransferase; ALB, albumin; Scr, serum creatinine; LDH, lactate dehydrogenase.
